# LiverWiki: a wiki-based database for human liver

**DOI:** 10.1186/s12859-017-1852-0

**Published:** 2017-10-13

**Authors:** Tao Chen, Mansheng Li, Qiang He, Lei Zou, Youhuan Li, Cheng Chang, Dongyan Zhao, Yunping Zhu

**Affiliations:** 1 Beijing Institute of Life Omics, State Key Laboratory of Proteomics, Beijing Proteome Research Center, National Center for Protein Sciences (Beijing), Beijing Institute of Radiation Medicine, 33 Life Science Park Rd, Changping District, Beijing, 102206 China; 2 School of Software and Electrical Engineering, Swinburne University of Technology, Melbourne, Victoria 3122 Australia; 3 Institute of Computer Science and Technology, Peking University, No.5 Yiheyuan Road Haidian District, Beijing, 100871 China

**Keywords:** Wiki-based database, Human liver, Community-driven sharing

## Abstract

**Background:**

Recent advances in omics technology have produced a large amount of liver-related data. A comprehensive and up-to-date source of liver-related data is needed to allow biologists to access the latest data. However, current liver-related data sources each cover only a specific part of the liver. It is difficult for them to keep pace with the rapid increase of liver-related data available at those data resources. Integrating diverse liver-related data is a critical yet formidable challenge, as it requires sustained human effort.

**Results:**

We present LiverWiki, a first wiki-based database that integrates liver-related genes, homolog genes, gene expressions in microarray datasets and RNA-Seq datasets, proteins, protein interactions, post-translational modifications, associated pathways, diseases, metabolites identified in the metabolomics datasets, and literatures into an easily accessible and searchable resource for community-driven sharing. LiverWiki houses information in a total of 141,897 content pages, including 19,787 liver-related gene pages, 17,077 homolog gene pages, 50,251 liver-related protein pages, 36,122 gene expression pages, 2067 metabolites identified in the metabolomics datasets, 16,366 disease-related molecules, and 227 liver disease pages. Other than assisting users in searching, browsing, reviewing, refining the contents on LiverWiki, the most important contribution of LiverWiki is to allow the community to create and update biological data of liver in visible and editable tables. This integrates newly produced data with existing knowledge. Implemented in mediawiki, LiverWiki provides powerful extensions to support community contributions.

**Conclusions:**

The main goal of LiverWiki is to provide the research community with comprehensive liver-related data, as well as to allow the research community to share their liver-related data flexibly and efficiently. It also enables rapid sharing new discoveries by allowing the discoveries to be integrated and shared immediately, rather than relying on expert curators. The database is available online at http://liverwiki.hupo.org.cn/.

## Background

Liver is one of the largest and most important organs in the human body. It is responsible for many critical functions in the human body. Its malfunction can cause significant damage to the human body. Due to its importance, research on liver and liver diseases focus on fully elucidating its functions with global analysis at the “omics” level, e.g., genomic, proteomic, transcriptomic, and metabolomic. Consequently, it fuels a rapid increase in the amount of liver-related data generated. It is a challenge to manage and integrate such rapidly and continuously generated data.

Many existing databases provide specific data about liver-related gene, gene products, gene expressions, pathways and liver diseases [1–6]. However, these data sources each cover only a specific part of the liver. It is very difficult for biologists to keep pace with the rapid increase in liver-related data. Some of those data sources are no longer updated or available due to the lack of proper maintenance caused by limited human resource and funding support. Although some databases are still being updated from time to time, they certainly cannot keep pace with or scale with the rapid increase in liver-related data. Thus, newly generated research data cannot be shared and transferred in a flexible and efficient manner.

Moreover, some communities that focus on liver-related research are too small to establish or maintain a liver-related data source. They usually reveal their new discoveries only by publications. As a result, a large body of liver-related data in scientific publications is waiting to be extracted and integrated into a proper data source. It is important that newly generated liver-related data can be rapidly and easily integrated with existing data for flexibly and efficient sharing in an accessible and searchable manner.

In order to allow biologists to keep pace with the continuously increasing liver-related data, a comprehensive and up-to-date source of information on liver-related genes, proteins, protein interactions, post-translational modifications, associated pathways and diseases is required. Integrating diverse liver-related data from all kinds of data sources is a formidable challenge that requires sustained human effort. Constructing and maintaining these data in a flexible and efficient manner is also challenging.

Fortunately, wiki-based biological databases have received a great deal of attention in recent years [7]. The idea of a wiki on gene function was first proposed based on the report that wikipedia comes close to Britannica in terms of the accuracy of its science entries [8]. Later on, there has been an significant increase in the construction domain-specific wiki-based databases [9–26]. However, none of the databases target human liver.

To address the above issues, we designed LiverWiki, the first wiki-based database for integrating liver-related genes, homolog genes, gene expressions in microarray datasets and RNA-Seq datasets, proteins, protein interactions, post-translational modifications, associated pathways, diseases, metabolites identified in the metabolomics datasets, and literatures for community-driven sharing. LiverWiki supports community searching, browsing, reviewing, refining, and creating liver-related data, which allow newly produced data to be rapidly integrated with existing data through community curation. Flexible internal links are provided to demonstrate the relations between genes, proteins, pathways and diseases. Powerful external links are used for direct access to external databases. The main goal of LiverWiki is to provide the research community with comprehensive liver-related data, as well as to allow the community to share their liver-related data flexibly and efficiently. It also allows small institutions to rapidly reveal their new discoveries by immediately integrating the discoveries into this easily accessible and searchable data source rather than relying on expert curators.

## Construction and content

In order to allow users to contribute and share comprehensive liver-related data collaboratively, we integrate diverse liver-related data obtained and mined from existing biological databases, experimental data from human liver proteomic plan (HLPP), and scientific publications. Specifically, liver-related genes were found mainly from NCBI-Gene [5]. The annotations from Gene Ontology are also collected for each gene. Summaries of gene-related diseases and gene-associated protein are also provided for each gene, if available. Liver-related homolog genes are also collected from NCBI-Gene [5]. Liver-related proteins were collected mainly from UniProtKB [6]. They are annotated by data imported from Gene Ontology [27].Other than liver-specification and significant expressions in hepatocellular carcinoma, validation of the protein in the Human Liver Proteome Project (HLPP) is provided if the protein is validated by HLPP experiments [28]. Protein-protein interactions (PPIs) are collected mainly from HAPPI [29] and Reactome [30], as well as the experimental results in the HLPP project. Post-translational modifications (PTMs) are imported from Phospho-ELM [31], PhosphoSitePlus [32] and HLPP project. Summaries of protein-related diseases and protein-associated gene are also provided for each protein, if available. Liver-related transcriptome data, including the Microarray datasets, RNA-SEQ datasets and gene expressions, are imported from GEO [33] and SRA [34]. Liver-related pathways are mainly imported from SMPDB [35]. The metabolomics data were retrieved from MetaboLights and Metabolomics Workbench whose metadata have been indexed by OmicsDI [36–38]. Liver diseases are imported from DO [1], and UMLS [39]. These liver diseases are organized by Human Liver Disease Ontology (HuLDO) developed by ourselves [40]. HuLDO is a standardized method to classify and annotate human liver diseases. It is a comprehensive lexicon which contains detailed information on hepatic disease and demonstrates the logical and medical relationships between different diseases [40]. To assess the quality of each entry, we use a semi-quantitative method which considers the reliability and the number of data sources. The curation of these data on LiverWiki pages is a useful starting point for users who want to contribute to LiverWiki.

### Content

LiverWiki houses information on over 141,897 content pages and category pages. Specifically, it includes 19,787 liver-related gene pages, 17,077 homolog gene pages, 50,251 liver-related protein pages, 36,122 gene expression pages, 2067 metabolites, 16,366 disease-related molecules, and 227 liver disease pages. It also contains 227 disease categories, 638 pathway categories, 37 transcriptome dataset categories (24 RNA-Seq datasets and 13 microarray datasets), 36 metabolomics datasets, and 62 relation categories to describe the relationships between liver disease and related molecules.

### Disease-centric page types

**Fig. 1:**
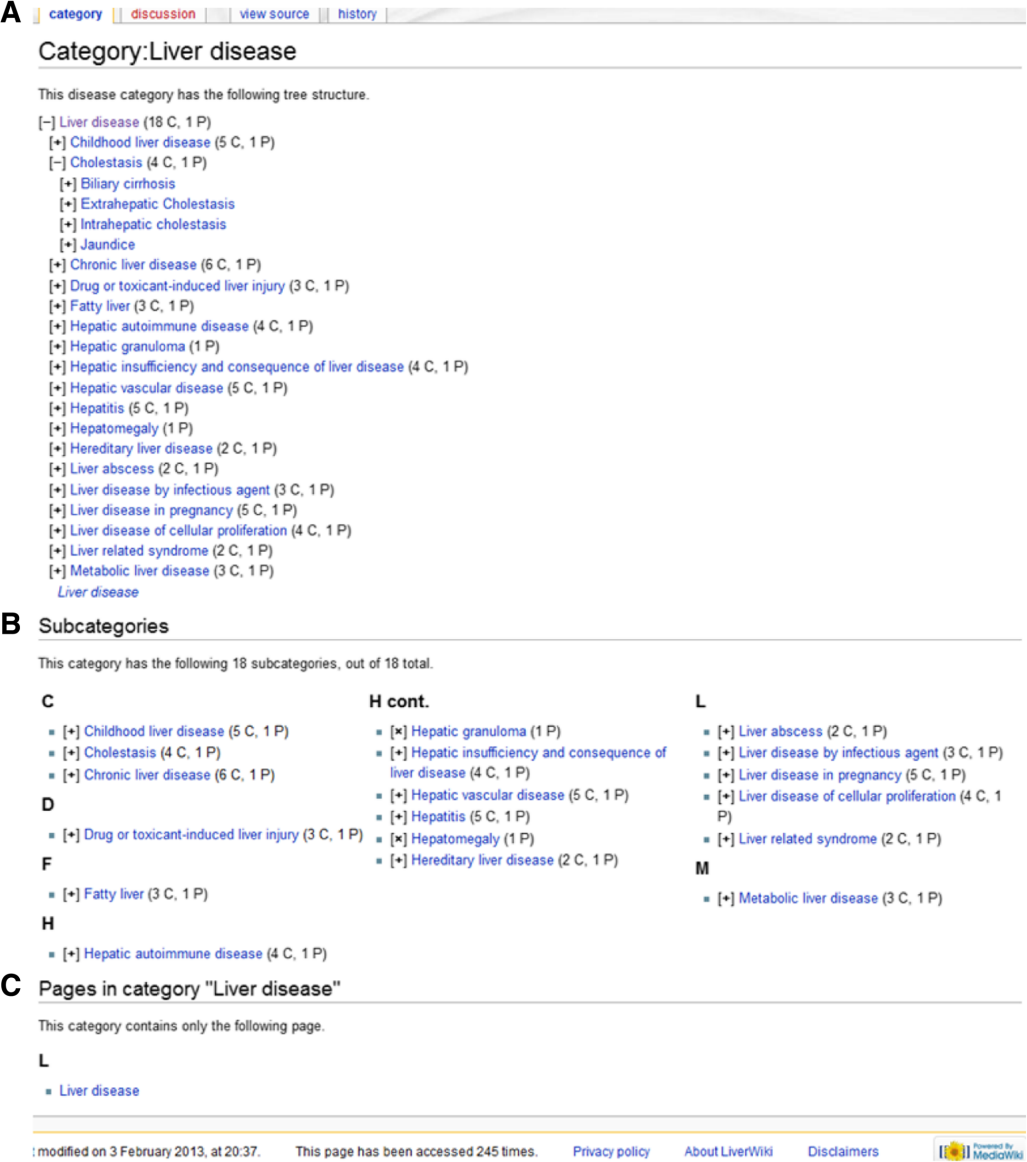
An example of disease category page for the disease term ‘Liver disease’. **a** Systematic tree view of Human Liver Disease Ontology (HuLDO), with a few nodes expanded to show the subcategories of a specific disease. **b** List view of this disease term ordering by first letter of alphabet. **c** Link to the content page of this disease term

**Fig. 2:**
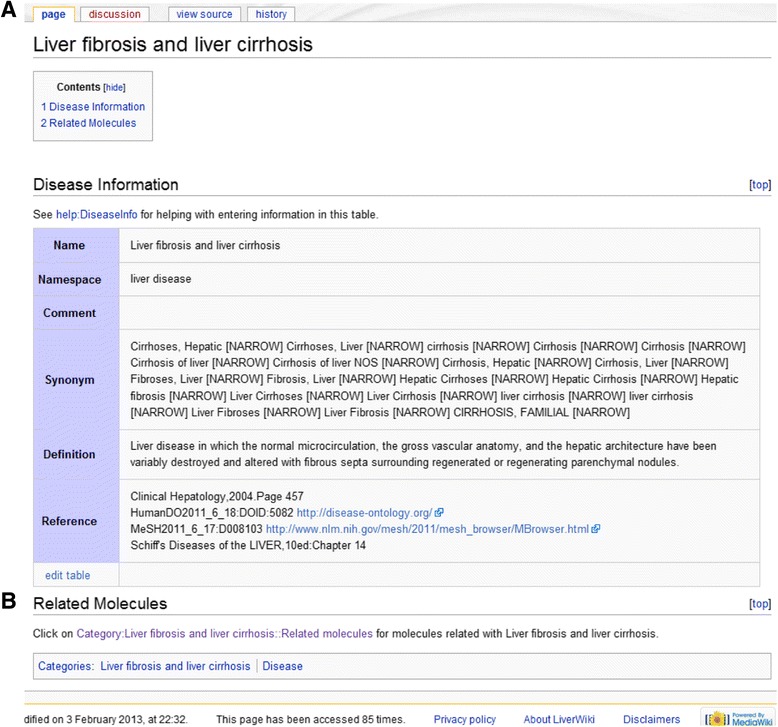
Content page of ‘Liver fibrosis and liver cirrhosis’. **a** Basic information of this disease term in editable table. **b** links to the list of relations between this term and related molecules

**Fig. 3:**
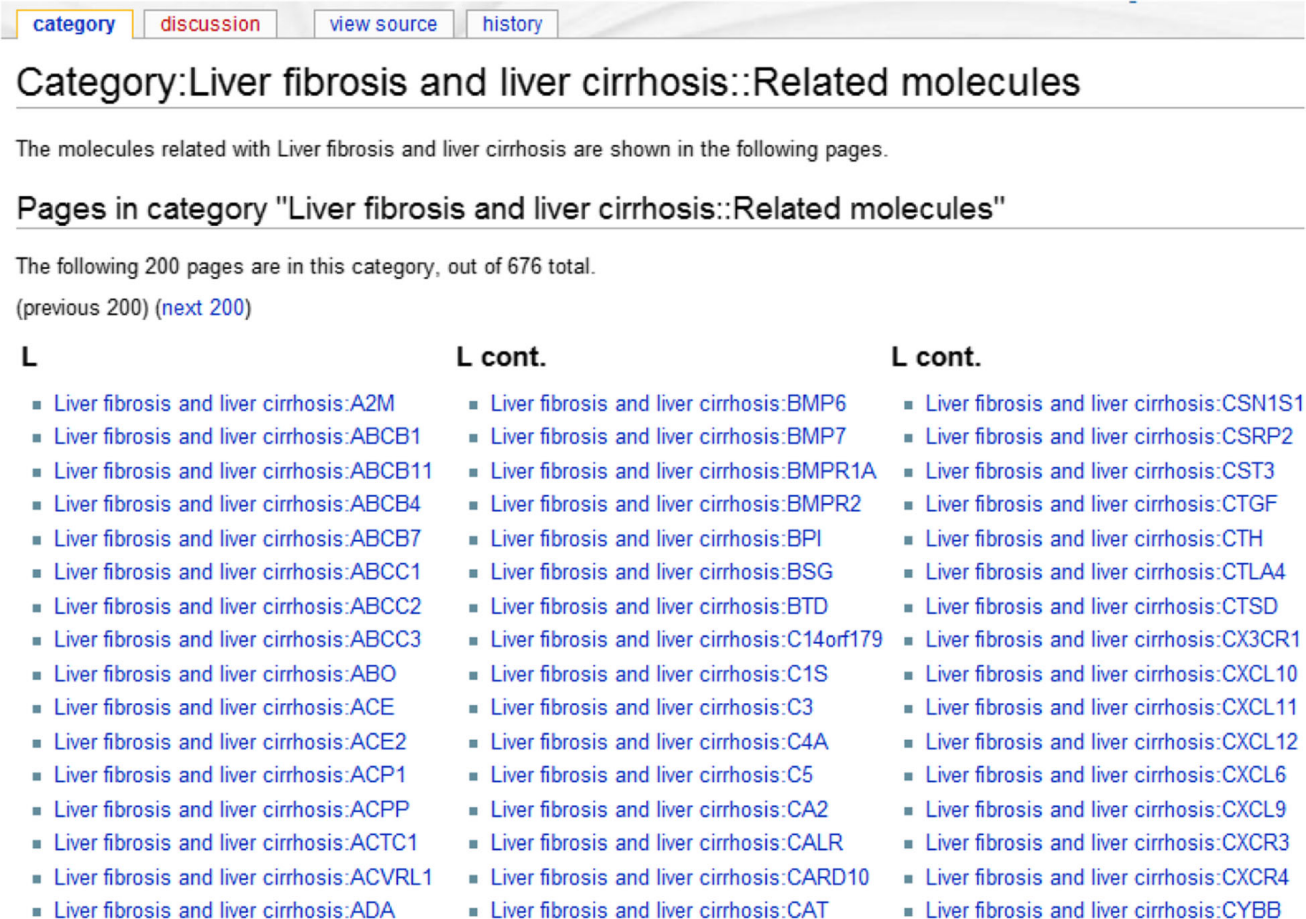
A typical page with a list of relations between the disease term ‘Liver fibrosis and liver cirrhosis’ and its related molecules. It lists all associated genes or proteins related with this disease term

LiverWiki contains 227 pages for 227 different kinds of human liver diseases. Disease terms from HuLDO are used as the basics for the page names. Disease terms from HuLDO are both represented by mediawiki category pages and content pages. Figure 1 shows an example of a category page for the term ‘Liver Disease’. This term can be considered as the root node of a subcategories of a specific term. Each term in the tree has links to their child term pages as subcategories, and links to the associated content page as a category member. For example, link on the term ‘Hepatitis’ in the tree takes users to the category page for Hepatitis in which the term ‘Hepatitis’ can be taken as the root of the sub-tree. Link on the category page of a disease term also takes the user to the content page of this term. The content page provides users with details of the disease in the form of tables. It includes name, namespace, comment, synonym, definition, and reference. An example of disease content page is shown in Fig. 2. On the content page, there is a link that takes user to the list of relations between this disease term and relevant molecules. Figure 3 shows an example of a page with a list of relations between a disease term and its relevant molecules.

### Other page types

Other than liver-related disease category page and content page, LiverWiki has the relationship page that describes the relationship between diseases and genes, or between diseases and proteins. The relationship page is named using ‘:’ to concatenate the disease term and the gene symbol, e.g., Hepatocellular carcinoma:ACE or the disease term and protein name, e.g., Hepatocellular carcinoma:1433B_HUMAN. It includes the disease name, phenotype, related molecule, type, detection method, change type, conclusion, reference, and confidence. Each page also includes links to the disease content page and molecule page. At the bottom of a relationship page is a category link to a page with a list of relationships between the specific disease and related molecules.

**Fig. 4:**
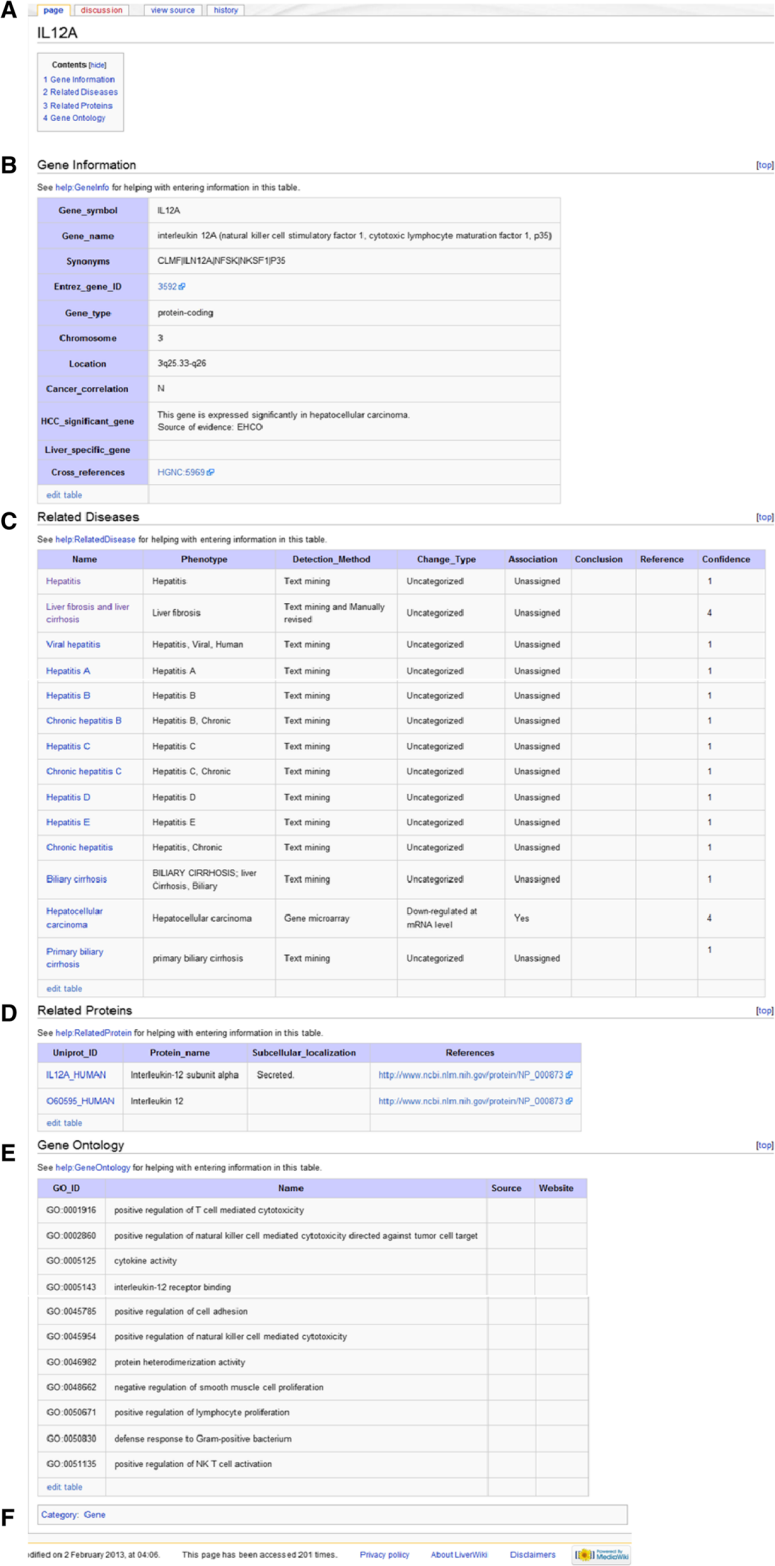
An example of gene page in LiverWiki. **a** Section list of the page. **b** Basic information of this gene in user-editable table. **c** The relations between this gene and its related diseases. Links on the disease term can take users to the disease content page. **d** Gene products. Clicking the link on the protein guides user to the protein page. **e** Gene ontology. **f** Category which this gene page belongs to

Gene symbols from NCBI [5] are used as the gene page names. A gene page provides users with the gene name, synonyms, Entrez gene ID, gene type, chromosome, location, cancer correlation, cross references, and annotations from Gene Ontology. Cross references include links which can be clicked for direct access to external databases. Summaries of gene-related diseases and proteins are also provided on the page. Links on theses disease terms and protein names will guide users to associated disease content pages and protein pages. Clicking the category link at the bottom of this page takes users to a list of all the genes on LiverWiki. Figure 4 presents an example of a gene page.

**Fig. 5:**
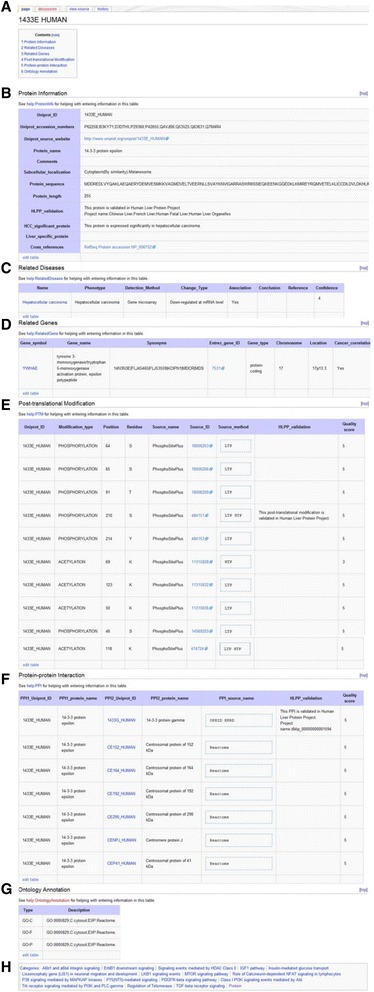
A typical protein page for 1433E_HUMAN in LiverWiki. **a** Section list of the page. **b** Basic information of this protein in user-editable table. **c** The relations between this protein and its related diseases. **d** Related Gene. **e** PPIs. The experimental information about PPI from HLPP is also provided. **f** PTMs. We also report the experimental data about PTM from HLPP. **g** Gene ontology. **h** Categories which this protein page belongs to

The protein page name uses a canonical entry name from Uniprot [6]. The protein page includes data about the Uniprot ID, accession numbers, source website, protein name, comment, subcellular localization, sequence, length, and cross references, PTMs, PPIs, as well as ontology annotations. The category link at the bottom of this page takes users to a list of all the proteins on LiverWiki. This page also reports the experimental data about the protein from HLPP. Figure 5 presents one of the protein pages as an example.

Both on the gene and protein pages, liver-related data and whether the gene or protein is significantly expressed in hepatocellular carcinoma are also provided.

Each homolog gene page includes the top 10 most relevant orthologous genes of species. The homolog gene page name uses ‘:’ to concatenate the gene symbols and the string ‘homolog’, e.g., IL12A:homolog. It provides users with gene symbols, gene IDs, description, locations, and aliases for the homolog genes. The transcriptome page contains information about the dataset and platform. It provides external URL links to the GEO data source. It also includes links to associated gene expression pages as category members. The gene expression page is named using ‘:’ to concatenate the gene symbol and transciptome dataset name. Each metabolomics page includes information about the dataset and metabolites identified in the dataset. The metabolite page is named using ‘:’ to concatenate the metabolite and the name of the metabolomics dataset. Pathways and literature pages are also provided on LiverWiki. Each pathway page is a category page with links to the associated protein pages as category members.

As on other mediawiki-based wikis, LiverWiki pages are paired with talk pages to support various discussions, commentary and questions. Each page also contains all the typical elements, including a sidebar and tabs along the top for various actions.

Other than disease category pages that show the tree structure of HuLDO, LiverWiki also uses other category pages to increase its usability for users. Users can place pages in corresponding categories, and subcategories in categories. A feature of LiverWiki is that users can create new category to reorganize the pages on LiverWiki. Table 1 lists the major types of content pages and category pages on LiverWiki.

**Table 1:** Major types of pages in LiverWiki

Page type		Number of pages
Content page	Disease	227
	Gene	19,787
	Homolog gene	17,077
	Protein	50,251
	Literature	1964
	Gene expressions	36,122
	Metabolite	2067
	Disease-Related molecules	16,366
Category page	Disease	227
	Pathway	638
	Microarray dataset	13
	RNA-Seq dataset	24
	Metabolomics dataset	36
	Gene	1
	Protein	1
	Related molecules	62
	Literature	1
	Total	144,864

Customized tables on each page are used to accommodate structured data. LiverWiki also provides hyperlinks to the source website for each term. Related disease and protein terms shown on gene pages are linked to corresponding disease and protein page. Similar links can be found on disease pages, protein pages, gene expression pages, pathway pages, transcriptome dataset pages, as well as relationship pages which demonstrate the relationships between diseases and their related-molecules.

Similar to other mediawiki-based wikis, LiverWiki pages are associated with talk pages that offer places for questions, comments and discussion.

LiverWiki also uses the category technology to improve its usability. At the moment, there are a total of 9 categories on LiverWiki, as shown in Table 1. Users can create new categories in addition to these 9 categories. The creation of new categories will be presented in the next section.

## Utility and discussion

LiverWiki integrates a variety of human liver-related data for community-driven sharing in an accessible and searchable manner. It supports community editing, creating, searching, or browsing, and enables rapid integration of newly generated data with existing data by community curation. Currently, we have curators that review the new pages/tables to ensure the accuracy of the information because the user group is relatively small at the moment. As the user group continues to grow, user participation is be included to ensure the accuracy of the information on LiverWiki following the wiki model: the quality of information is ensured and improved by multiple users reviewing and refining the same content [21]. When the user group grows bigger, pages/tables created by users are to be reviewed by peers in co-editing manners to ensure the accuracy of the information on the pages/tables.

Data updating method and frequency: We have developed a standard pipeline to retrieve and parse data updates from other sources through APIs provided by NCBI, GEO, OmicsDI, etc. The retrieval and parsing of the data, as well as the update of the data on LiverWiki, are carried out automatically. We have also developed a literature mining tool named MedCurator (available at http://medqrator.hupo.org.cn/MedQRator) to retrieve liver-related data from scientific publications. Thus, articles covered in PubMed can be automatically updated on LiverWiki.

### User-editable table on every page

Editable table is one of the key components on each page. Those editable tables can be updated by users to refine their contents. Anonymous users can browse and search LiverWiki. However, registration is required for users to edit the tables on the pages. The link “Edit table” at the bottom of each table takes registered users to the editing page where they can edit the content in the table. They can also add new columns. This is very convenient for users to correct existing errors and to provide data that supports or refutes existing page contents. Each page is also associated with a talk page that supports questions, comments and discussions.

The collaborative nature of LiverWiki allows users to contribute collaboratively to LiverWiki. Help documents about editing new pages are available via the ‘help’ menu on the left sidebar of the home page.

### User-editable new page

**Fig. 6:**
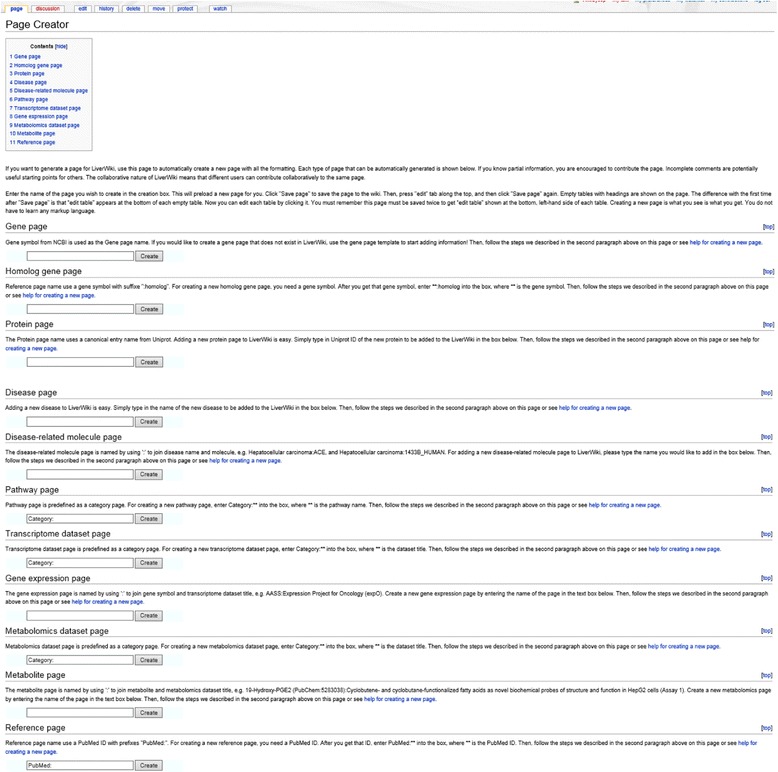
Creators for 11 kinds of pages, including gene page, homolog gene page, protein page, disease page, relationship page, pathway page, microarray dataset and RNA-Seq dataset page, gene expression page, metabolomics dataset page, metabolite page and reference page

One of LiverWiki’s most important features is the flexible creation of new pages. Users can create new pages using pre-defined templates. LiverWiki offers 11 types of page creators for registered users, which are shown in Fig. 6. Clicking the ‘Page Creator’ link on the homepage will take users to the page where they can create new pages. After entering the new page name in the form, clicking on the ‘create’ button triggers a script that generates an editable version of the new page and preloads it with a pre-defined table template. Empty and editable tables with headings are presented on the page. Users can also create new pages without using any of the 11 in-built templates. Visually editable tables offer guidance for users during the process for adding data in customized formats. Category is used not only for automatically organizing pages into categories, but also for dynamically creating the relationships between terms, such as diseases and genes.

### Other functionalities

A user-friendly web interface is provided for users to easily search or browse LiverWiki. Users can search LiverWiki by entering keywords in search box on the sidebar of each page. LiverWiki supports page title search and full-text search regardless of the order of the search keywords. The “Go” button on the left sidebar will direct user to the corresponding page the title of which matches the search keywords; otherwise, the search engine will return a list of pages the contents of which match the search keywords. Upon a click on the ‘Search’ button on the left sidebar, LiverWiki returns a list of pages to the user. For example, a search with keywords ‘Liver fibrosis and cirrhosis’ will return matched pages, including ‘Liver fibrosis and liver cirrhosis’, ‘Liver fibrosis and liver cirrhosis caused by hepatitis’, ‘Liver fibrosis and liver cirrhosis:A2M’, ‘A2M’, etc. The result list includes the diseases, relationships between the diseases and their molecules, the molecules, etc. The link on each result takes users directly to the corresponding page. The same pages are returned as the result to searches with the same keywords in different orders, e.g., ‘Liver fibrosis cirrhosis’ or ‘liver cirrhosis fibrosis’. Search engine is order-insensitive and case-insensitive. Similarly, users can use the search keywords relevant to genes, proteins, pathways, gene expressions or relationships between disease and its related molecules. Furthermore, advanced search is provided for all types of namespaces, such as talk, category, template and so on.

All categories can be browsed in a tree view or list view. In order to display the relationships between liver diseases, a tree view is applied. Links in the tree view takes users to the sub-tree view of the chosen disease term. List views ordered by alphabetically are used for other terms.

Registration is available on the right top corner of each page. This is mainly to inhibit vandalism. To control user groups, LiverWiki employs a ‘vampire model’ for user registration. Only registered users can create new accounts. In an academic setting, trust of peers is relatively high. A single account can be created for a principal investigator who can create accounts for their students. This approach for account creation relieves the burden of having to create all accounts from a single user. Version control is provided to handle erroneous editing by rolling back to an earlier version of the page.

LiverWiki handles complex data with structured tables. Structured tables allow users to modify the contents which can be extracted without the need for natural language processing. External links are provided to navigate to specific pages on external public databases. Internal links guides users to build relationship between internal terms.

### Database implementation

LiverWiki was created using the mediawiki technology that powers wikipedia, allowing users to contribute on many different levels. Java program is developed to post the seed data onto LiverWiki using mediawiki’s application program interface (API). LiverWiki is constructed on two major layers, namely data layer and user interface layer. The former is implemented with a MySQL relational database and the latter is driven by mediawiki and Apache Tomcat running on a Linux server. LiverWiki utilizes mediawiki’s extensions to implement the search interface, the tree-structure display, the table edition function, spam prevention, and so on.

## Conclusions

LiverWiki is the first wiki that provides comprehensive data about human liver and relevant topics, e.g., liver diseases and liver-related genes. It was established to integrate a variety of high-quality data about genes, gene expressions in microarray datasets and RNA-Seq datasets, homolog genes, proteins, their interactions, PTM, metabolites identified in the metabolomics datasets, associated pathways and diseases, with cited peer-reviewed scientific publications. It provides a user-friendly web interface for users to search, browse, refine, review existing contents or create new contents. It can be continuously updated by the community to keep pace with the rapid increase in liver-related data. The availability of the data can be used to better understand liver and study relevant diseases that are important to human health.

Small organizations that are unable to maintain a database can use LiverWiki to rapidly share their new discoveries of liver-related data. They can easily publish their new data on this accessible and searchable data resource so that it can be shared immediately within the community. The development of LiverWiki is tightly coordinated with the HLPP project.

LiverWiki is very user-friendly. We believe that LiverWiki is a valuable database, which will provide significant support for researchers and practitioners in the field of liver research.

## Abbreviations

DO: Disease ontology
GEO: Gene expression omnibus
HAPPI: Human annotated and predicted protein interaction database
HLPP: Human liver proteomic plan
HuLDO: Human liver disease ontology
NCBI: National center for biotechnology information
OmicsDI: Omics discovery index
PPIs: Protein-protein interactions
PTM: Post-translational modification
PTMs: Post-translational modifications
SMPDB: The small molecule pathway database
UMLS: Unified medical language system
Uniprot: Universal protein resource
